# UGT74D1 Is a Novel Auxin Glycosyltransferase from *Arabidopsis thaliana*

**DOI:** 10.1371/journal.pone.0061705

**Published:** 2013-04-16

**Authors:** Shang-Hui Jin, Xin-Mei Ma, Ping Han, Bo Wang, Yan-Guo Sun, Gui-Zhi Zhang, Yan-Jie Li, Bing-Kai Hou

**Affiliations:** 1 The Key Lab of Plant Cell Engineering and Germplasm Innovation, Education Ministry of China, Jinan, Shandong Province, P. R. China; 2 School of Life Science, Shandong University, Jinan, Shandong Province, P. R. China; Wake Forest University, United States of America

## Abstract

Auxin is one type of phytohormones that plays important roles in nearly all aspects of plant growth and developmental processes. The glycosylation of auxins is considered to be an essential mechanism to control the level of active auxins. Thus, the identification of auxin glycosyltransferases is of great significance for further understanding the auxin regulation. In this study, we biochemically screened the group L of *Arabidopsis thaliana* glycosyltransferase superfamily for enzymatic activity toward auxins. UGT74D1 was identified to be a novel auxin glycosyltransferase. Through HPLC and LC-MS analysis of reaction products in vitro by testing eight substrates including auxins and other compounds, we found that UGT74D1 had a strong glucosylating activity toward indole-3-butyric acid [IBA], indole-3-propionic acid [IPA], indole-3-acetic acid [IAA] and naphthaleneacetic acid [NAA], catalyzing them to form corresponding glucose esters. Biochemical characterization showed that this enzyme had a maximum activity in HEPES buffer at pH 6.0 and 37°C. In addition, the enzymatic activity analysis of crude protein and the IBA metabolite analysis from transgenic *Arabidopsis* plants overexpressing *UGT74D1* gene were also carried out. Experimental results indicated that over-production of the UGT74D1 in plants indeed led to increased level of the glucose conjugate of IBA. Moreover, *UGT74D1* overexpression lines displayed curling leaf phenotype, suggesting a physiological role of UGT74D1 in affecting the activity of auxins. Our current data provide a new target gene for further genetic studies to understand the auxin regulation by glycosylation in plants.

## Introduction

Auxin is the first discovered phytohormone and is well-known for its regulatory role in virtually all aspects of plant growth and development, such as general root and shoot architecture, organ initiation and patterning, cell division and differentiation [1]–[3], plant responses to biotic and abiotic stresses, *etc* [4]–[5]. Auxins belong to chemically diverse compounds, most of which have an aromatic system such as indole, phenyl or naphthalene ring with a side chain containing a carboxyl group attached. The hormone is known to exist as the free acid or in conjugation with a wide variety of compounds such as amino acids, peptides, and sugars [6].

Indole-3-acetic acid (IAA) is the most abundant auxin natively generated in plants and its *in vivo* role has been examined extensively. However, there are other native endogenous auxins in plants. Indole-3-butyric acid (IBA) has been identified in a number of plant species such as maize (*Zea mays*), pea (*Pisum sativum*) and *Arabidopsis*, comprising approximately 25% to 30% of the total free auxin pool in *Arabidopsis* seedlings [7]. It is reported that the concentration of free IBA is comparable to the level of free IAA in a number of plants [8]. As for IBA *in vivo* functions, many studies support the idea that IBA only functions as a precursor of IAA. For example, IBA can be converted to IAA in a process similar to fatty acid β-oxidation and the IBA-to-IAA conversion enzymes have been identified [9]–[14]. In addition, many Arabidopsis mutants unable to convert IBA to active IAA have reduced root growth sensitivity to IBA, but normal sensitivity to IAA, suggesting that IBA is an essential auxin precursor rather than active auxin [13]–[17]. However, it is also argued in other literatures that IBA may have activity independent of IAA. For example, the activity of IBA can affect lateral root induction, adventitious root initiation, elongation of roots, shoots, and hypocotyls [18]–[20], as well as the induction of auxin-responsive genes [21]–[22]. In a recent report, the increase of free IBA, but not IAA level in UGT74E2OE plants indicated that the shoot morphogenesis might be directly affected by IBA rather than its conversion to IAA [23]. This partial independence of both auxins was further supported by accounted differences in IAA and IBA polar transport [18], [19], [24], [25].

The hormonal homeostasis is defined as “the maintenance of a steady state concentration of the hormones in the receptive tissue appropriate to any fixed environmental condition” [6]. Plants use several mechanisms to control the level of endogenous auxins. Despite the regulation of synthesis and degradation of these phytohormones, plants may store auxins in the form of conjugates [26]. IAA and IBA can be conjugated via amide linkage to amino acids such as aspartate and by ester linkages to glucose or myo-inositol [27]–[29]. The level of conjugated forms of IAA and IBA may be higher than their free forms and are considered to be inactive storage and/or transport forms of the hormone [29]–[30]. Their enzymatic release to free forms is an important part of auxin metabolism. For IBA, its ester conjugates dominate over amide forms of IBA. Moreover, IBA conjugates are more easily hydrolyzed and more slowly transported in different plant systems, perhaps leaving more phytohormones at the plant base in comparison with conjugates of IAA [9], [29], [31]. In addition, certain IBA conjugates are very active in bioassays [9], [27]. The formation and hydrolysis of auxin conjugates is developmentally regulated and varies significantly among plant tissues [32].

Glucose conjugates of both IBA and IAA have been identified in plants, including *Arabidopsis* [9], [31], [33]. Glycosyltransferases catalyze the addition of sugar to auxins. Since glycosylation can alter many properties of the aglycones in respect to their bioactivity, solubility, as well as their cellular localization, glycosylation is considered as an important regulatory mechanism for the cellular homeostasis and activity of phytohormones [34]–[35]. So far, several auxin glycosyltransferases have been identified from plants. *iaglu* is the first auxin glycosyltransferase gene identified from *Zea mays* [36]. UGT84B1 and UGT74E2 were then identified from *Arabidopsis* and chemically demonstrated that they have high activity toward auxins, particularly toward IAA and IBA, respectively [23], [37]. Three related enzymes (UGT84B2, UGT75B1, and UGT75B2) were also identified with trace activities [37]. When overexpressing UGT84B1 or UGT74E2 in *Arabidopsis*, a clear disturbance in auxin homoestasis and obvious growth defects were observed [23], [38]. These findings suggest that auxin glycosyltranferases are important players for auxin activity and plant development. In addition, the existence of multiple glycosyltransferases toward the same type of phytohormone in one plant species may implicate a synergistic effect of multiple glycosyltransferase members beneficial for plant evolution and adaptation.

**Figure 1:**
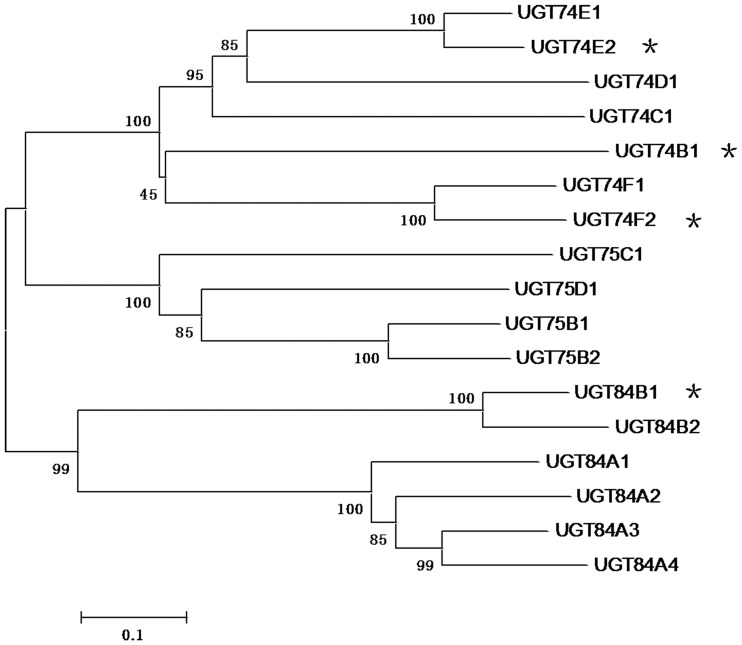
Phylogenetic relationship of the group L glycosyltransferases from *Arabidopsis thaliana*. The phylogenetic tree of *Arabidopsis* UGTs is adopted from the previous report [40]. Bootstrap values are indicated above the nodes. The glycosyltransferase sequences were retrieved from Carbohydrate-active enzymes database (http://www.cazy.org/GT1_eukaryota.html) and NCBI database. The asterisks indicate those glycosyltransferases with confirmed enzymatic activities toward phytohormone related compounds.

Our research interest is to screen phytohormone-related glycosyltransferases from *Arabidopsis*. Till now, 120 UDP-glycosyltransferase (UGT) have been identified and classified into 14 groups in the *Arabidopsis* genome [39]–[40]. Among them, group L becomes our first target because several hormone-related UGTs, including UGT84B1 and UGT74E2, were identified from this group (Figure 1). In our screening, UGT74D1 was identified to be a novel auxin glycosyltransferase, but we cannot exclude the possible activity for other members. We provide in this study solid evidence to show the enzyme activity and biochemical characterization of UGT74D1. Moreover, a metabolites analysis of auxin glucosyl-conjugates and phenotypic analysis for the *UGT74D1* overexpressing transgenic plants were carried out as well.

## Materials and Methods

### Chemicals

Most of the substrates used in this study were purchased from Sigma-Aldrich (St. Louis, MO USA). UDP-Glucose was purchased from Meryer (Shanghai, China). Glutathione-coupled Sepharose 4B beads and reduced form glutathione were obtained from Amersham Pharmacia (Piscataway, NJ USA). Restriction enzymes, ligation enzymes and PrimeSTAR HS DNA Ploymerase were purchased from TaKaRa (Shiga, Japan).

### Cloning, Plasmid Construction and Sequence Analysis of *UGT74D1*

Standard DNA manipulation techniques were used. Full-length cDNA of *UGT74D1*(At2g31750) was amplified from *Arabidopsis* by reverse transcription-PCR (RT-PCR) with a pair of primers, UGT74D1-a: 5′-CGCCATATGGGAGAGAAAGCGAAAGC-3′ and UGT74D1-b: 5′-CCGCTCGAGTTACCTCACAATTTTAGC-3′, which contain restriction sites at 5′ terminals for *Nde*I and *Xho*I, respectively. The PCR product was cloned by recombination into pBluescriptSK. In order to obtain a prokaryotic expression vector with suitable and multiple restriction sites, pGEX-2T vector was modified according to methods described by Zhang and co-authors [41] with a slight modification. The modified pGEX-2T vector has the multiple clone sites *Bam*HI, *Nde*I, *Not*I, *Sph*I, *Nco*I, *Sal*I, *Sac*I, *Xho*I, *Hin*dIII, *Eco*RI and is designated as pGEX-3H. *UGT74D1*cDNA was subcloned from pBluescript SK plasmid into pGEX-3H between the sites of *Nde*I and *Xho*I to obtain the expression plasmid of GST-UGT74D1 fusion protein.

The phylogeny of 17 gene which were in the L group of *Arabidopsis* family 1 glycosyltransferases were obtained from the alignments using ClustalX 2 and Neighbor–Joining trees constructed with bootstrap sampling of 1000 replications using MEGA 4.0 programs. The *Arabidopsis* UGT sequences used in the phylogenetic tree were obtained from the Carbohydrate-active enzymes database (http://www.cazy.org/GT1_eukaryota.html) and the NCBI database.

### Protein Putification and Enzyme Activity Assay

*Escherichia coli* strain XL1-Blue carrying the expression plasmid of GST-UGT74D1 fusion construct was used to produce the fusion protein. Soluble recombinant protein was induced and purified according to the methods described by Hou et al. [42]. Protein concentration of the eluted fractions was determined with Coomassie Protein Assay Reagent (Thermo Scientific) using bovine serum albumin as reference. The purified recombinant fusion protein was also analyzed by SDS-PAGE following the methods described by Sambrook et al [43].

The glycosyltransferase activity assay was carried out following the conditions described by Tognetti and co-workers with modifications [23]. The assay mix (100 µl) contained 2 ug of purified UGT74D1 fusion protein, 5 mM UDP-glucose, 1 mM hormone, 50 mM HEPES (pH 7.0), 2.5 mM MgSO_4_, 10 mM KCl and 14.4 mM 2-mercaptoethanol. The reactions were carried out at 37°C for 3 h and then stopped by the addition of 10 µl of trichloroacetic acid (240 mg/ml), quick-frozen, and stored at −20°C before reverse-phase HPLC analysis.

### HPLC and LC/MS Analysis

20 µl of each sample was loaded by means of a auto sampler SIL-20A (Shimadzu HPLC system equipped with the diode array detector SPD-M20A, the SIL-20A, the commuications bas module CBM-20A, the degasser DGU-20A3 and the workstation LC solution), onto a 5 µm C18 column (150×4.6 mm; Welch, Ultimate). A linear gradient with increasing methanol (solvent A) against distilles H_2_O (solvent B) at a flow rate of 1 ml/min over 30 min was used to separate the glucose conjugates from their aglycones. Both solutions contained 0.01% H_3_PO_4_. Each peak on the chromatogram was monitored between 190 and 430 nm.

The HPLC conditions were described in the following: IAA, λ_detection_ = 210 nm, 10%–48% solvent A; ICA, IPA, IBA, and NAA, λ_detection_ = 280 nm, 10%–70% solvent A; 2,4-dichlorophenoxyacetic acid and picloram, λ_detection_ = 287 nm, 10%–100% solvent A.

The products of auxin conjugates synthesized by recombinant UGT74D1 were further confirmed by the LC-MS system (Thermo Scientific) including the Surveyor autosampler and MS pump (Thermo-Finnigan, San Jose, CA, USA). The methods and mobile phases were similar to HPLC condition except that 0.01% acetic acid instead of 0.01% H_3_PO_4_. The mass spectrometer operated in a positive electrospray ionization mode with 30 eV and a probe voltage of 3.0 kV. The temperature was set to 350°C. The data acquisition and analysis were performed with Xcalibur software (version 2.0.6).

### Factors Affecting the Activity of Recombinant UGT74D1

Because UGT74D1 has the highest enzyme activity toward IBA, we choose the IBA in this study as substrate for analyzing the factors affecting the enzyme activity. The calculation of enzyme activity was based on the reduction of peak area of the substrate IBA before and after reaction. Factors tested include temperature, buffer and pH. All the reaction mix (100 µl) contained 0.2 ug of recombinant UGT74D1, 5 mM UDP-glucose, 1 mM IBA, 2.5 mM MgSO_4_, 10 mM KCl, 14.4 mM 2-mercaptoethanol. For the temperature test, 50 mM HEPES (pH7.0) was added and the reactions were performed at four different temperature values (20°C, 30°C, 37°C and 45°C). For the buffer and pH test, 50 mM Tris buffer (pH 6.0–9.0), 50 mM HEPES buffer (pH 5.0–9.0), 50 mM MES buffer (pH 5.0–9.0) or 50 mM phosphate buffer (pH 6.0–9.0) was added and the reactions were performed at 37°C. All the reactions were carried out for 30 min and then stopped by adding 10 µl trichloroacetic acid (240 mg/ml), quick-frozen, and stored at −20°C before reverse-phase HPLC analysis.

### Assays of Glycosyltransferase Activity and Glucosylated Metabolites of Auxin in Transgenic Plants

The full-length cDNA of the *UGT74D1* gene was subcloned from the pBluescriptSK into the plant overexpression vector pBI121 and replaced the glucuronidase (GUS) gene. The overexpression construct was transferred into *Agrobacterium tumefaciens* GV3101 and then transformed into *Arabidopsis* (Col-0) via floral dip method [44]. At least four homozygous transgenic lines were selected by kanamycin resistance and the overexpression of *UGT74D1* was determined by RT-PCR.

Total crude protein was extracted from 2-week-old transgenic seedlings as described previously [42]. To investigate the glycosyltransferase activity of the crude protein extracts prepared from plant tissues, 50 µl crude protein extracts (containing ∼0.1 mg of total protein) were mixed 1 mM auxin, 5 mM UDP-glucose, 50 mM HEPES (pH7.0), 2.5 mM MgSO_4_, 10 mM KCl, and 14.4 mM 2-mercaptoethanol, in a 100 µl reaction. The reactions were incubated at 37°C for 1 h and were stopped by the addition of 10 µl of trichloroacetic acid (240 mg/ml). The reaction mix was analyzed subsequently using reverse-phase HPLC following the method described above.

To analyze the amount of the glucose conjugates of interest in the transgenic plants, the wild-type and *UGT74D1* transgenic plants (line 23, line 24) were grown on the MS agar plates for 12 days, and removed carefully to immersed in MS liquid culture system with or without 100 µM IBA. After incubation for 24 h, 1 g of plant tissues from each line was collected, frozen in liquid nitrogen, and stored at −80°C prior to the extraction. The extraction of IBA glucose conjugates was carried out following the method described previously [42]. 0.1 mM picloram was added as internal control at the beginning of the extraction to monitor the recovery rate. The amounts of IBA glucose conjugates in extraction buffers of different transgenic lines were analyzed by HPLC as described above.

### Leaf-flattening Experiments of Transgenic Plants

*Arabidopsis* plants were grown in the greenhouse on Nutrition Soil (Shangdao Biotech Co. Ltd., Shandong, China) with vermiculite (Nutrition Soil:vermiculite = 2∶1) at 22±2°C under a 16/8 h light/dark cycle with a light intensity of ∼100 µmol m^–2 ^s^–1^. When plants reached growth stage ∼6.5 after growing for 5 weeks, the lamina of the representative seventh rosette leaf was detached from the petiole and the flattening index was calculated according to the method described by de Carbonnel et al. [45].

## Results

### Purification of Recombinant UGT74D1

**Figure 2:**
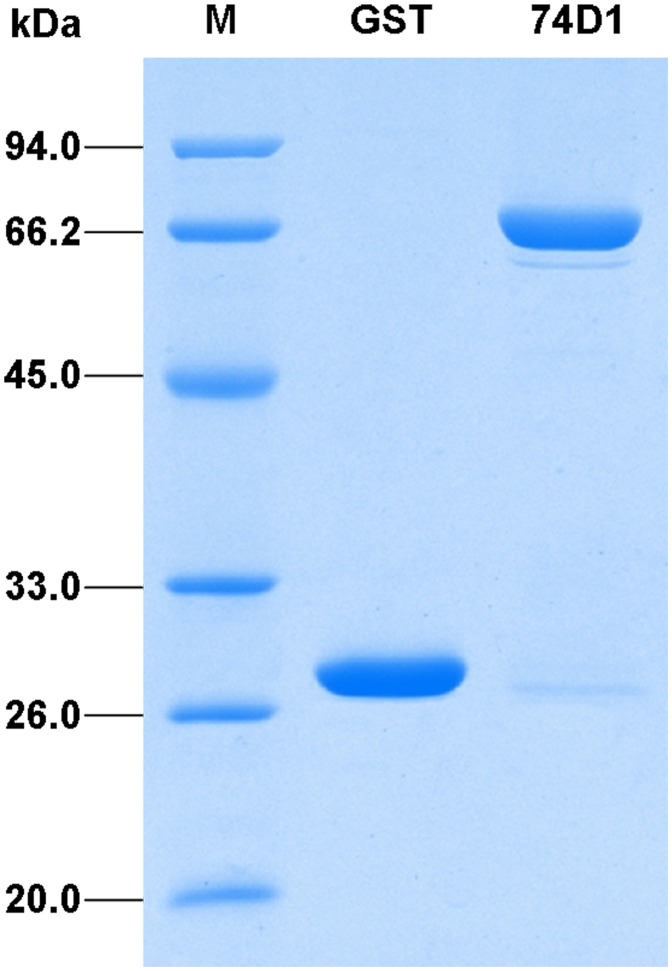
SDS-PAGE analysis of the recombinant GST-UGT74D1 fusion protein. Proteins were purified from *E.coli*, analyzed on a 12% (w/v) polyacrylamide gel and visualized with Coomassie Brilliant Blue staining. M: protein molecular weight marker; GST: glutathione-s-transferase; 74D1: fusion protein.

In order to explore more hormone-related UGTs, in this study, we put our focus on other members of group L whose activity and substrate have not been previously demonstrated. These UGTs were cloned into prokaryotic expression vector and expressed in *Escherichia coli* tagged with glutathione *S*-transferase (GST). *UGT74D1* gene is predicted to encode a protein of 456 amino acid residues with a theoretical molecular weight of 50.2 kDa, thus the recombinant fusion protein should be 76.2 kDa together with GST tag. The SDS-PAGE analysis showed that the molecular mass of the purified fusion protein was between 66.2 kDa and 94.0 kDa, which was consistent with the theoretical prediction (Figure 2).

### Identification of UGT74D1 Enzymatic Activity Toward Auxins

**Figure 3:**
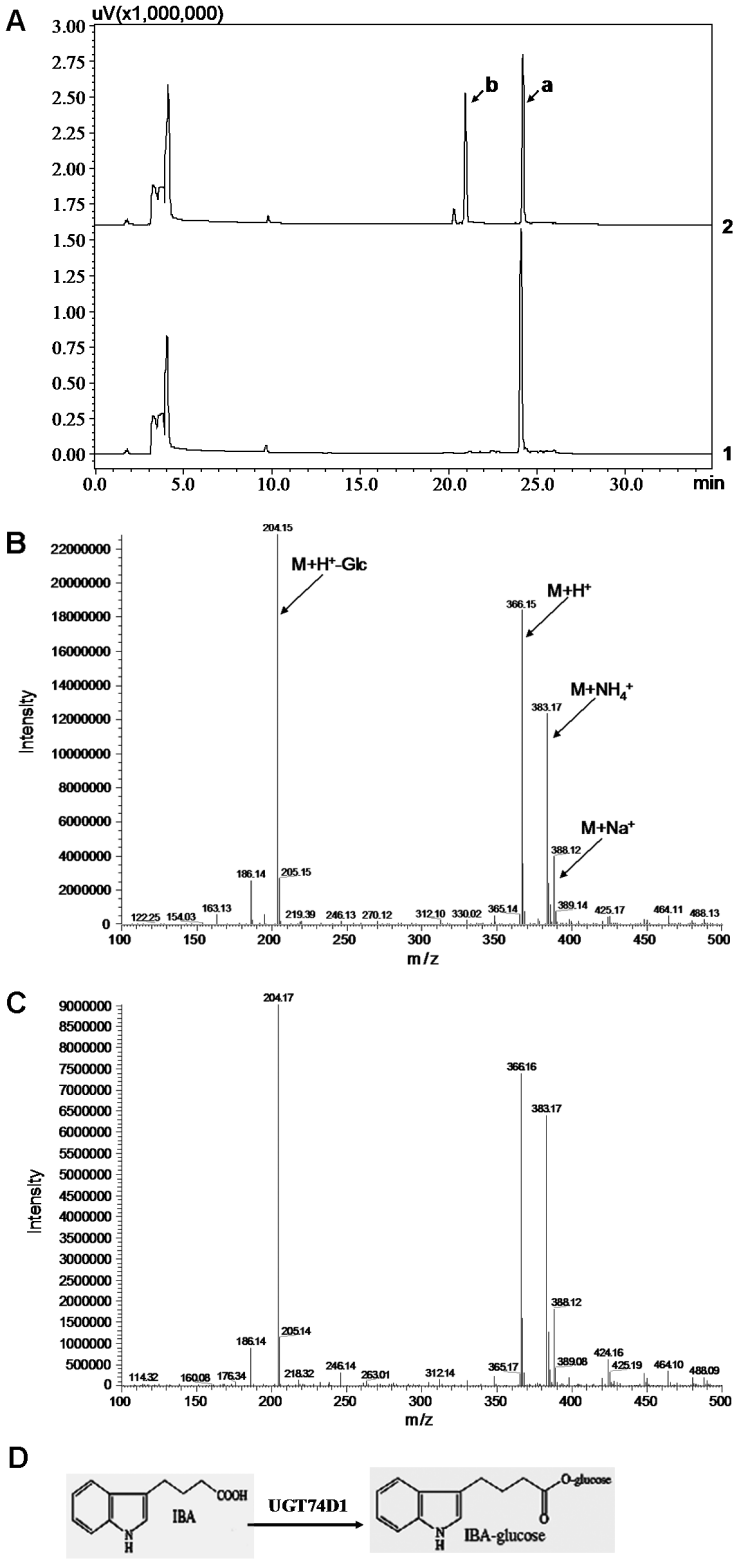
HPLC and LC-MS analysis of reaction product from IBA. (A) HPLC analysis. 1: the reaction was added with GST protein as control. 2: the reaction was added with UGT74D1 fusion protein and a new peak (peak b) was produced. Peak “a” represents the substrate IBA. (B) LC-MS analysis of peak b. (C) LC-MS analysis of IBA glucose conjugates produced by the catalysis of UGT74E2 which was used as positive control in this research. (D) Proposed enzymes and biosynthetic pathway for the synthesis of IBA-glucose ester from the aglycone IBA.

Using UDP-glucose as the sugar donor, the recombinant UGT74D1 was tested *in vitro* for its glycosyltransferase activity against each of the seven substrates used in this study: indole-3-carboxylic acid [ICA], indole-3-acetic acid [IAA], indole-3-propionic acid [IPA], indole-3-butyric acid [IBA], the synthetic auxin analogs naphthaleneacetic acid [NAA], 2,4-dichlorophenoxyacetic acid [2,4-D], and picloram. The structures of these compounds used are listed in Figure S1. The following HPLC analysis and the recognition of new product peaks showed that the recombinant UGT74D1 had a strong activity toward IBA (Figure 3A). Therefore, we further conducted a LC-MS analysis to the new products. As shown in Figure 3B, in the positive ionization mode, putative IBA-glucose ester (IBA-Glc) gave a dominant ions *m/z* 204.15 (M+H^+^-glucose); *m/z* 366.16 (M+H^+^); *m/z* 383.17 (M+NH_4_^+^) and *m/z* 388.12 (M+Na^+^) (MW of IBA-Glc is 365.00). The mass spectrum peaks of putative IBA-Glc were identical to the peaks of a product catalyzed by UGT74E2 [23], which was demonstrated to be IBA-Glc and used as a positive control in our research (Figure 3C). Thus, a new biosynthetic pathway of IBA-glucose ester from the aglycone IBA by UGT74D1 catalysis was proposed (Figure 3D).

**Figure 4:**
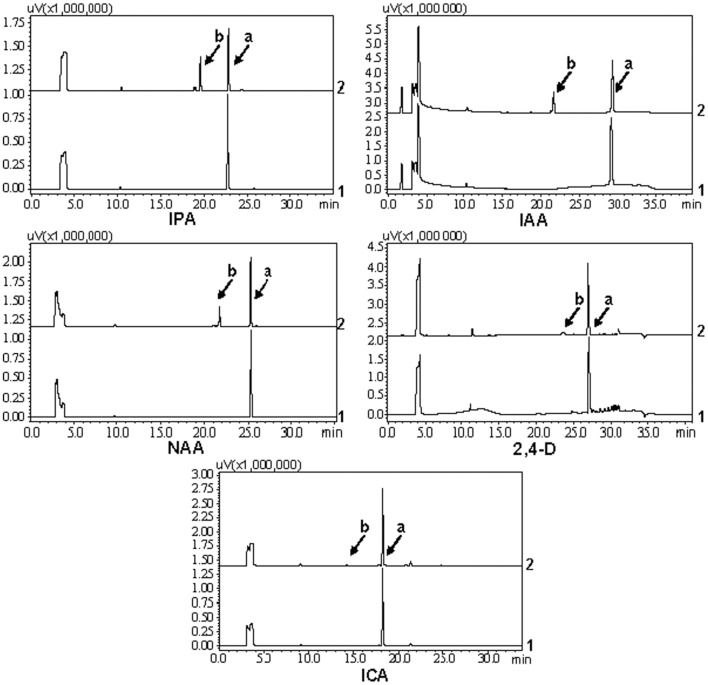
HPLC analysis of reaction products from other auxins. 1: the reaction was added with GST protein as control. 2: the reaction was added with UGT74D1 fusion protein. Peak “a” represents the auxin substrates. Peak “b” represents the reaction products.

As shown in Figure 4, UGT74D1 also had a significant activity toward other auxins with similar structure to IBA, for example, IPA, IAA and NAA, only a trace activity toward 2,4-D and ICA, whereas no activity toward picloram. The specific enzyme activities of UGT74D1 towards different substrates were also calculated (Table 1), and the data indicated that UGT74D1 was an auxin glycosyltransferase with the highest activity towards IBA.

**Table 1:** Specific activity of UGT74D1 toward auxins and related substrates.

Substrates	Specific activity (nkat/mg protein)
ICA	0.17±0.03
IAA	1.25±0.01
IPA	1.85±0.01
IBA	2.17±0.05
NAA	1.15±0.01
2,4-D	0.32±0.08
Picloram	ND

Note: The assay mix (100 µl) contained 2 ug of purified UGT74D1 fusion protein, 5 mM UDP-glucose, 1 mM hormone, 50 mM HEPES (pH 7.0), 2.5 mM MgSO_4_, 10 mM KCl and 14.4 mM 2-mercaptoethanol. The reactions were carried out at 37°C for 3 h. The results represent the means ±S.D from three independent measurements. The specific enzyme activity was defined as nmol of substrates converted into glucose conjugates per second (nanokatal, nkat) by 1 mg of protein.

The retention time (Rt) and λ_max_ of the glucose conjugates produced were as follows: ICA conjugate, Rt = 14.2 min, λ_max_ = 280 nm; IAA conjugate, Rt = 22.0 min, λ_max_ = 210 nm; IPA conjugate, Rt = 19.7 min, λ_max_ = 280 nm; IBA conjugate, Rt = 21.3 min, λ_max_ = 280 nm; NAA conjugate, Rt = 21.7 min, λ_max_ = 280 nm; 2,4-D conjugate, Rt = 24.0 min, λ_max_ = 287 nm.

### Biochemical Characterization of UGT74D1

**Figure 5:**
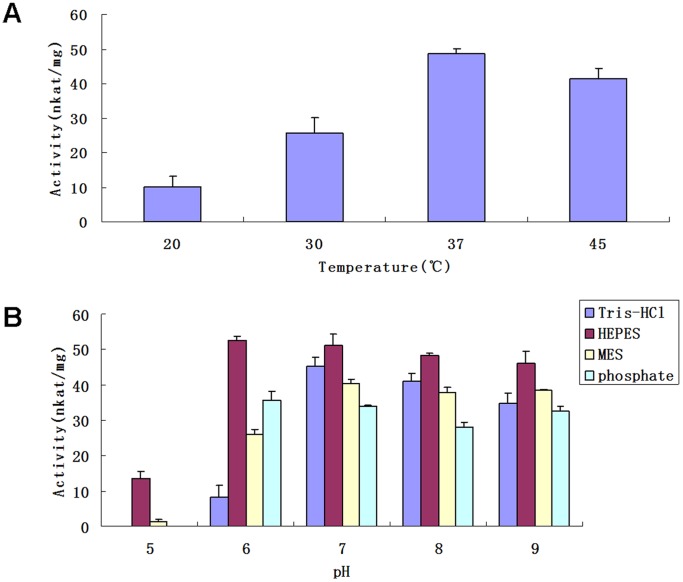
Analyses on factors affecting the activity of recombinant UGT74D1. (A) The effects of temperature. (B) The effects of buffer and pH value. All the reaction mix (100 µl) contained 0.2 ug of recombinant UGT74D1, 5 mM UDP-glucose, 1 mM IBA, 2.5 mM MgSO_4_, 10 mM KCl, 14.4 mM 2-mercaptoethanol, 50 mM buffer and was incubated for 30 min as described in “Materials and Methods ”. The results represent means±SD from three replicates. The specific enzyme activity was defined as nmol of substrates converted into glucose conjugates per second (nanokatal, nkat) by 1 mg of protein.

The results in Figure 5 summarize the effects of reaction conditions, including temperature, pH and buffer, on the catalytic activity of UGT74D1. Four temperature points were tested and the results showed that 37°C was the best (Figure 5A). The pH analysis using Tris-HCl buffer, HEPES buffer, MES buffer, and phosphate buffer showed that UGT74D1 was active over a broad pH range but with a maximum in HEPES buffer at pH 6.0 (Figure 5B).

### Enzyme Activity Analysis of Transgenic *Arabidopsis* Plants Overexpressing *UGT74D1*

**Figure 6:**
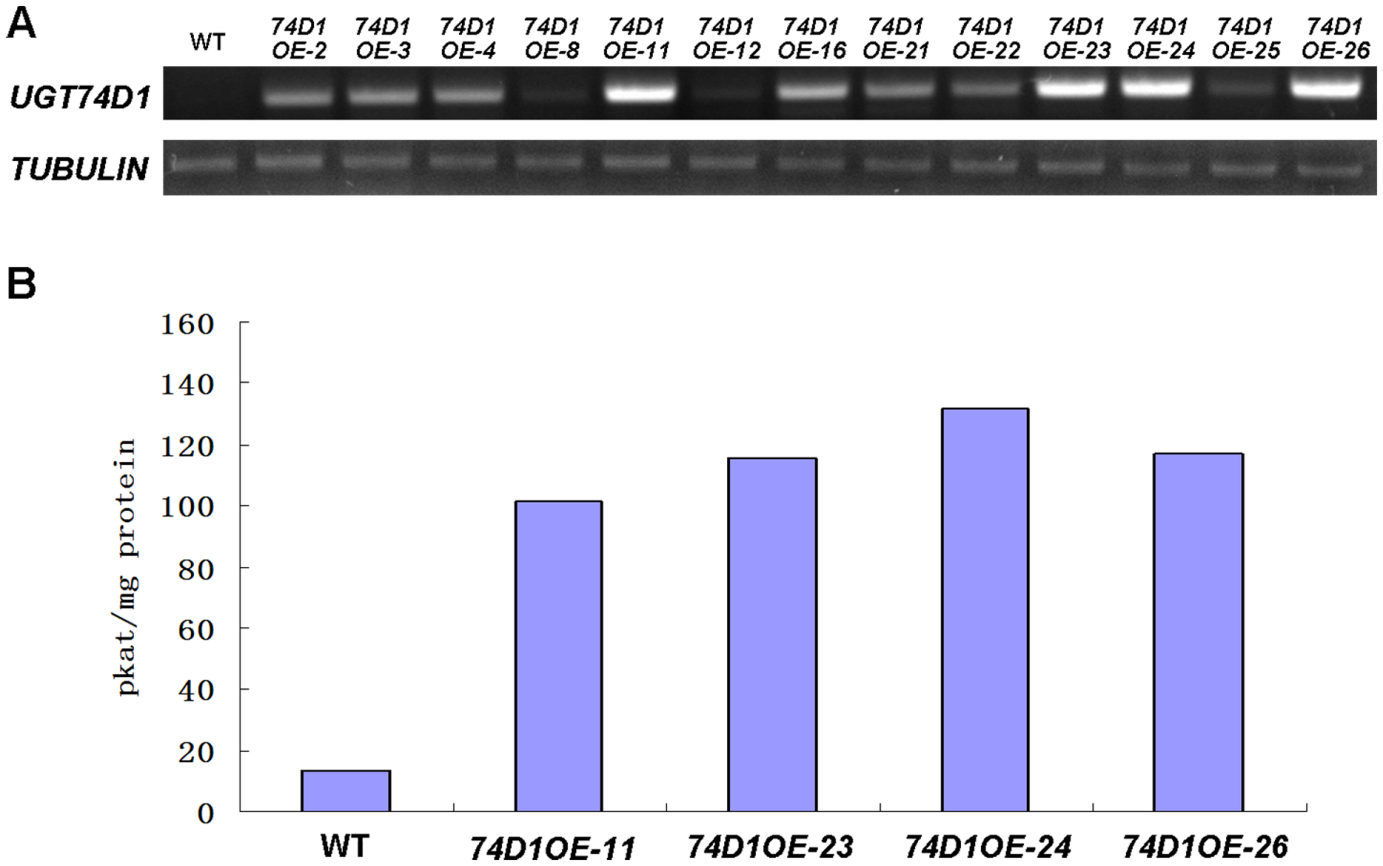
Analysis of the transgenic *Arabidopsis* plants overexpressing *UGT74D1* using the CaMV35S promoter. (A) RT-PCR analyses of the steady-state level of *UGT74D1* mRNA in transgenic plants (OEs) and wild type (WT). (B) The glycosyltransferase activities in the crude protein extracts of transgenic plants and wild type were measured following the procedure described under “Materials and Methods”. The specific enzyme activity was expressed as pmol of IBA glucosylated to form IBA-Glc by 1 mg of protein per second of reaction time at 37°C.

To gain further insights into the UGT74D1 activity, the transgenic plants overexpressing UGT74D1 driven by cauliflower mosaic virus 35S (CaMV35S) promoter were generated, and ten independent homozygous lines were obtained. As shown in Figure 6A, higher steady-state UGT74D1 level was observed in transgenic lines than that in wild-type plants. Seedlings of four transgenic lines were analyzed for enzyme activity toward IBA (Figure 6B). The results demonstrated that lines with higher *UGT74D1* transcripts also displayed stronger enzyme activity than wild type to form IBA-glucose conjugates.

### Glucosylated Metabolite Analysis of Transgenic *Arabidopsis* Plants

**Figure 7:**
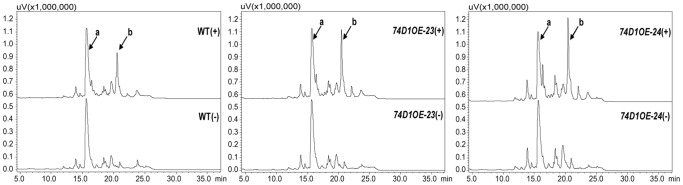
HPLC trace of IBA glucose conjugates of the extracts from the wild type (WT) and transgenic plants (OEs). (+) and (−) represent the plant tissues incubated with or without IBA before extraction process. Peak “a” indicates the picloram added as an internal control at the beginning of the extraction process; Peak “b” indicates the IBA-glucose conjugates. The extracts were analyzed with a linear gradient of methanol in H_2_O from 10–70% (all solutions contained 0.01% H_3_PO_4_) over 30 min at 1 ml/min and monitored at 280 nm.

To see whether the glucosidic metabolite is altered by enhanced expression of *UGT74D1*, exogenous IBA is applied to the transgenic and WT plants. As shown in Figure 7, if the plant tissues were not incubated with IBA before extraction process, IBA-glucose conjugates were below the level that could be detected or reliably quantified in our HPLC analysis. Upon application of IBA, however, considerable level of IBA-glucose conjugates were observed in both WT and transgenic plants. Compared to the amount of IBA-glucose produced in WT plants (130.81 pmol/mg.FW), much higher level of IBA-glucose in transgenic lines 74D1OE-23 (200.51 pmol/mg.FW) and 74D1OE-24 (277.55 pmol/mg.FW) can be detected. These data indicated that over-production of the UGT74D1 in the plants indeed led to increased level of the glucose conjugate of IBA.

### Phenotypes of Transgenic *Arabidopsis* Plants

**Figure 8:**
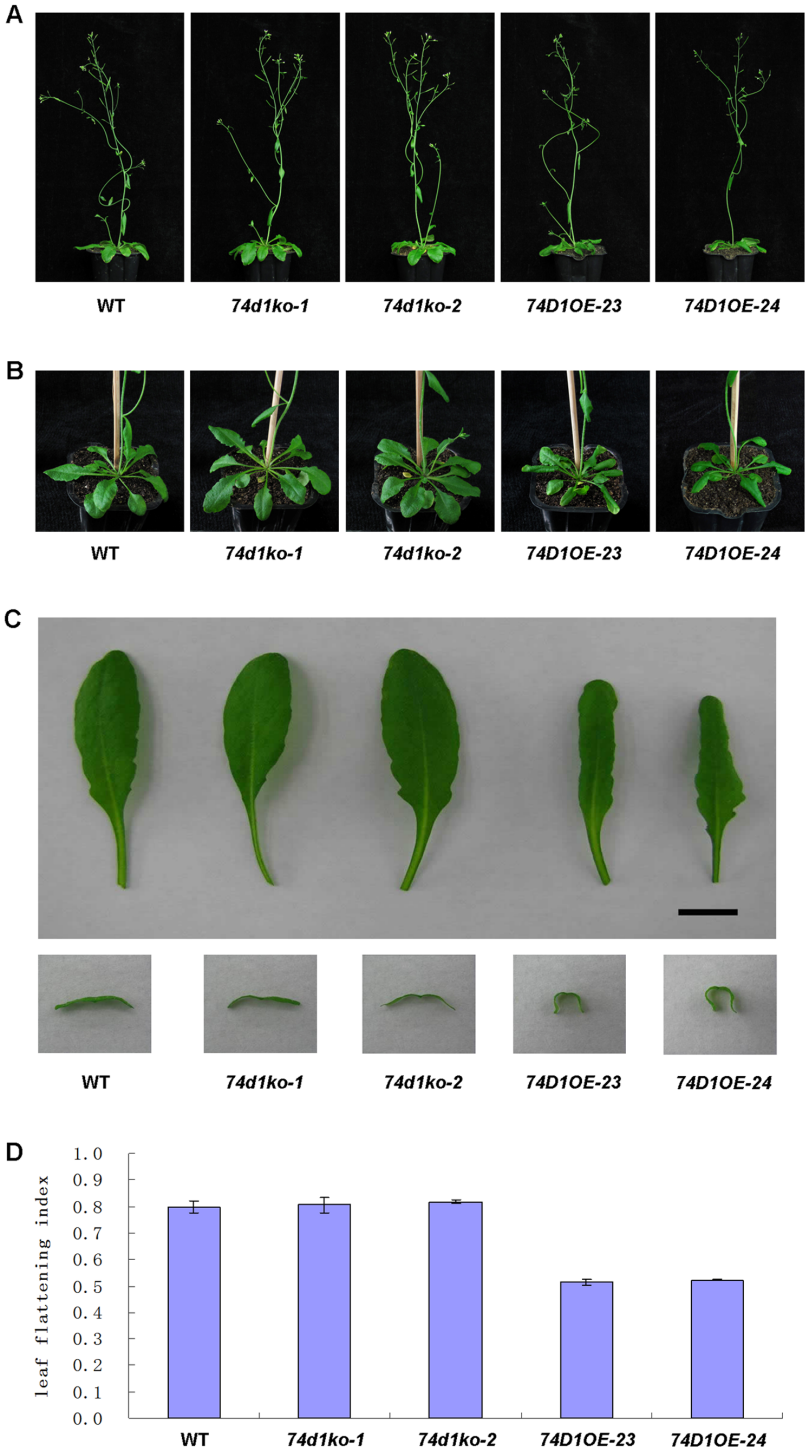
Phenotypes of Transgenic *Arabidopsis* Plants. (**A**) 5-week-old plant phenotypes of WT, mutants and overexpression lines. (B) 5-week-old rosette leaf phenotypes of wild type, mutants and overexpressor lines. (C) Seventh leaf and leaf transverse section of 5-week-old plants. (D) The flattening index of seventh leaf was calculated by dividing the projection area of intact curled leaves with that of manually uncurled leaves.

Two knockout mutants, *74d1ko-1* (Salk_004870) and *74d1ko-2* (Salk_011286), were confirmed to have no UGT74D1 transcripts (data not shown). Two transgenic lines over-expressing UGT74D1, *74D1OE-23* and *74D1OE-24*, were also confirmed (Figure 6). Preliminary observation indicated that, although homozygous knockout plants and overexpression lines had the similar phenotypes with wild-type including shoot height, shoot branching and root gravitropism (Figure 8A, Table 2), UGT74D1OE plants displayed curling leaves that differed from those of the wild-type plants at flowering stage (Figure 8B, 8C, 8D). The curling leaf phenotype of UGT74D1OE plants began to emerge after growing for four weeks, but was even more pronounced after growing for five weeks (growth stage ∼6.5), suggesting a physiological role of UGT74D1 in affecting the activity of auxins in leaves at this developmental stage.

**Table 2:** Phenotype comparison of overexpression lines of *UGT74D1*, *UGT84B1* and *UGT74E2* genes.

Overexpression lines	UGT74D1	UGT84B1	UGT74E2
Curling leaf	curling	wrinkly and curling	no
Compressed rosette	no	compressed	compressed
Shorter stature	no	shorter	shorter
Shoot branching	unchanged	higher level	higher level
Root gravitropism	unchanged	reduced	unchanged
Osmotic stress tolerance	not detected	not detected	increased

## Discussion

Glycosylation is a widespread physiological phenomenon, and is thought to be one of the most important mechanisms in maintaining plant cell homeostasis [34]. Glycosyltransferases are the enzymes responsible for glycosylation. They can typically transfer single or multiple activated sugars from nucleotide sugar donors, especially UDP-glucose, to a wide range of small molecular acceptors, thus change their bioactivity, solubility, stability, subcellular localization and binding properties. A detailed phylogenetic analysis classified the *Arabidopsis* family1 glycosyltransferases into 14 groups (A–N) based on their sequence homology and pattern of intron gain [39]. Several members of group L have been identified to glucosylate plant compounds to form their glucose esters [23], [37], [46]–[48]. In this study, we provide solid evidence that UGT74D1 of group L is a novel glycosyltransferase that can catalyze auxin glycosylation. Our results lay the groundwork for future genetic approaches in better understanding the regulation of auxin homeostasis by glycosylation in plants.

The data present here showed that UGT74D1 had different enzyme activities toward different auxins: IBA>IPA>IAA>NAA>2,4-D>ICA. It appears that the substrate preference of UGT74D1 might result from its regioselectivity to substrates and the side chain length of auxins which plays a major role in determining the glucosylating activity, thus the highest activity of UGT74D1 is with IBA and the lowest is with ICA. As yet, the relationship of these activities to events within the plants is unknown. Although IBA is the preferred substrate for UGT74D1 *in vitro*, the enzyme may glucosylate both IAA and IBA *in planta* depending on cell specificity of the enzyme expression, relative availability of substrates, and relative compartmentation of the enzyme and substrates.

Up to now, there has been several master glycosyltransferases identified from *Arabidopsis* to be responsible for the auxin glycosylation which include UGT84B1 mainly toward IAA [37], UGT74E2 and UGT74D1 mainly toward IBA [23, and this research]. These findings suggest that plant evolution has involved the formation of functionally redundant multiple glycosyltransferases toward the same type of phytohormones. It is therefore possible that additional glycosyltransferases may exist in *Arabidopsis* that are capable of glucosylating auxins. Why do functionally redundant auxin glycosyltransferases exist in plants? A synergistic or coordinated effect between different glycosyltransferase members may be meaningful for the fine tuning of auxin homeostasis. On the other hand, the spatial-temporal expression patterns of these genes might be different, which may have the potential to enhance the plant flexibility in development or in the adaptation to diverse environments.

It was reported that the constitutive expression of *UGT84B1* or *UGT74E2* in *Arabidopsis* resulted in many features typical of auxin-deficient phenotypes [23], [38]. These findings indicated the significance of those auxin glycosyltransferases in maintaining normal growth and development of plants. However, it appears that those auxin glycosyltransferases do not have exactly the same role. For example, *UGT84B1* and *UGT74E2* overexpresssors displayed the same phenotypes in compressed rosette, shorter stature and more shoot branches. On the other hand, *UGT84B1* overexpressors also had wrinkle leaves and reduced root gravitropism, but *UGT74E2* overexpresssors don’t (Table 2). Our observations in this present study on transgenic lines indicate that UGT74D1 has an influence on leaf growth, resulting in curling leaves of transgenic plants, but no other phenotypes were observed. Thus, the data described suggest that UGT74D1 is a novel auxin-UGT and has specific effects, providing a new target gene for further genetic study of auxin activity and regulation. Through further analyses of cell- and environment-specific expression of UGT74D1, followed by detailed metabolite profiling of auxins, we would get more insights into its *in vivo* substrates, its physiological impact on auxin homeostasis and even its possible synergistic effect with other auxin glycosyltransferases.

## Supporting Information

Figure S1 **The molecular structures of auxins used in this study as substrates for the enzymatic activity identification of UGT74D1.**
(TIF)
