# Correction: UGT74D1 Is a Novel Auxin Glycosyltransferase from *Arabidopsis thaliana*

**DOI:** 10.1371/annotation/457d7567-fc12-421c-9d79-880950ab10e1

**Published:** 2013-08-21

**Authors:** Shang-Hui Jin, Xin-Mei Ma, Ping Han, Bo Wang, Yan-Guo Sun, Gui-Zhi Zhang, Yan-Jie Li, Bing-Kai Hou

A grant was incorrectly added in the Funding Statement. The first sentence of the Funding Statement should read: "This research was supported by grants from the National Natural Science Foundation of China (No. 91217301 to B.K.H.)." 

