# Supplementary figures and images for: UGT74D1 Is a Novel Auxin Glycosyltransferase from Arabidopsis thaliana
Source: PLoS One. 2013 Apr 16;8(4):e61705. doi: 10.1371/journal.pone.0061705 (PMC3628222; doi:10.1371/journal.pone.0061705)

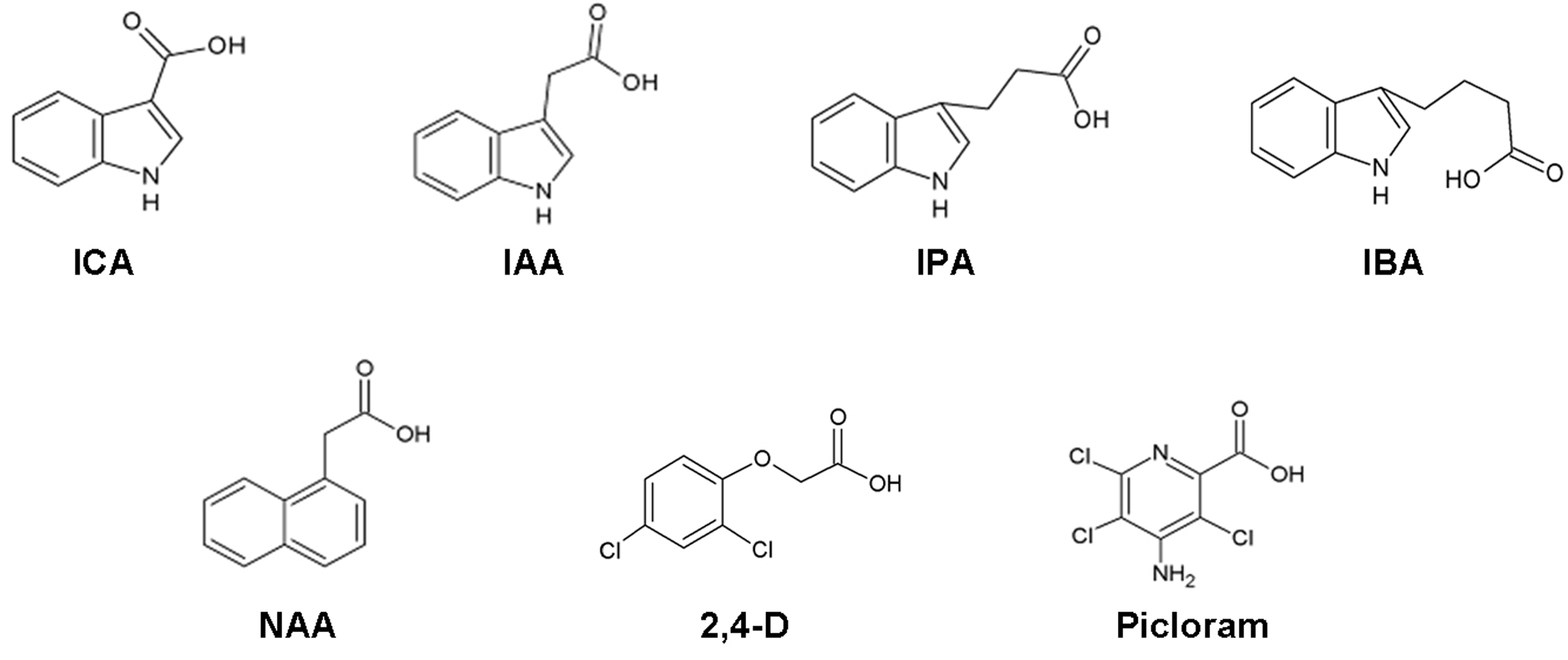

Supplement: Figure S1 — The molecular structures of auxins used in this study as substrates for the enzymatic activity identification of UGT74D1. (TIF) [file pone.0061705.s001.tif]
